# Application of three-dimensional computed tomography bronchography and angiography in thoracoscopic anatomical segmentectomy of the right upper lobe: A cohort study

**DOI:** 10.3389/fsurg.2022.975552

**Published:** 2022-09-20

**Authors:** Mingbo Wang, Huilai Lv, Tao Wu, Wenda Gao, Yang Tian, Chunyue Gai, Ziqiang Tian

**Affiliations:** ^1^ Department of Thoracic Surgery, The Fourth Hospital of Hebei Medical University, Shijiazhuang, China; ^2^ Operating Room, The Fourth Hospital of Hebei Medical University, Shijiazhuang, China; ^3^ Department of Thoracic Surgery, Hangzhou TCM Hospital Affiliated to Zhejiang Chinese Medical University, Hangzhou, China

**Keywords:** ground-glass nodules, anatomical segmentectomy, three-dimensional computed tomography bronchography and angiography, lung neoplasms, thoracoscopy

## Abstract

**Objective:**

Three-dimensional computed tomography bronchography and angiography (3D-CTBA) can provide detailed imaging information for pulmonary segmentectomy. This study aimed to investigate the safety and effectiveness of 3D-CTBA guidance of anatomical segmentectomy of the right upper lobe (RUL).

**Methods:**

This was a retrospective analysis of anatomical segmentectomy of the RUL at the Thoracic Surgery Department of the Fourth Hospital of Hebei Medical University from December 9, 2013, to June 2, 2021. Preoperatively, all patients underwent contrast-enhanced CT of the chest (to determine the size of the pulmonary nodule) and a lung function test. 3D-CTBA has been performed since 2018; patients with vs. without 3D-CTBA were compared. Segmentectomy was performed according to nodule location.

**Results:**

Of 139 patients (46 males and 93 females, aged 21–81 years), 93 (66.9%) completed single segmentectomy, 3 (2.2%) completed single subsegmentectomy, 29 had combined subsegmentectomy, 7 had segmentectomy combined with subsegmentectomy, and 6 had combined resection of two segments. Eighty-five (61.2%) patients underwent 3D-CTBA. 3D-CTBA cases had decreased intraoperative blood loss (67.4 ± 17.6 vs. 73.1 ± 11.0, *P* = 0.021) and shorter operation time (143.0 ± 10.8 vs. 133.4 ± 20.9, *P* = 0.001). 3D-CTBA (Beta = −7.594, 95% CI: −12.877 to −2.311, *P* = 0.005) and surgical procedure (Beta = 9.352, 95% CI: 3.551–15.153, *P* = 0.002) were independently associated with intraoperative blood loss. 3D-CTBA (Beta = −13.027, 95% CI: −18.632 to 17.422, *P* *< *0.001) and surgical procedure (Beta = 7.072, 95% CI: 0.864–13.280, *P* = 0.026) were also independent factors affecting the operation time.

**Conclusion:**

Preoperative use of 3D-CTBA to evaluate the pulmonary vessels and bronchial branch patterns of the RUL decreased blood loss and procedure time and so would be expected to improve the safety and effectiveness of thoracoscopic segmentectomy.

## Introduction

In recent years, as high-resolution multidetector computed tomography (CT) has gained popularity, the diagnosis of pulmonary nodules is increasing, especially when CT screening is performed in patients with high-risk factors for lung cancer (1). So, understanding and research into pulmonary lesions, known as ground-glass nodules, has gradually deepened. These are nodules with a slightly increased density that do not obscure the underlying bronchial structures or vascular margins on high-resolution CT (2). The majority of small ground-glass nodules do not progress, but some are preinvasive non-small-cell lung cancer (NSCLC) associated with a favorable prognosis if treated early (3).

The preferred surgical treatment for lung cancer used to be lobectomy, but for small, minimally invasive lesions, most clinicians prefer less radical resection to help preserve lung function (4, 5). Limited resection techniques for the lung include wedge resection and segmentectomy (4). Although segmentectomy is more complex than wedge resection, it has shown similar oncological outcomes for patients with early-stage NSCLC (6). Segmentectomy can ensure that there is enough distance between the lesion and the surgical margin, thereby reducing tumor recurrence and improving long-term survival (7). In the Clinical Practice Guidelines on NSCLC (Version 5, 2017) developed by the National Comprehensive Cancer Network (NCCN), minimally invasive segmentectomy has been recommended as a surgical procedure for lung cancer (8).

Successful and safe segmentectomy requires insight into the individual anatomical changes of pulmonary arteries, veins, and bronchi. Therefore, preoperative modeling is one of the most important tools to ensure the success of thoracoscopic segmentectomy (9). Three-dimensional CT bronchography and angiography (3D-CTBA) uses computer analysis of CT images to convert two-dimensional image data into 3D geometric images (10). 3D-CTBA is the commonly used preoperative modeling technique in clinical practice and has been widely used in various clinical disciplines. In thoracic surgery, 3D-CTBA is used to reconstruct 3D images of vessels, bronchi, and tumors of target pulmonary lobes. Patients undergoing thoracoscopic segmentectomy or lobectomy with preoperative 3D-CTBA have a lower incidence of postoperative complications and a significantly shorter operation time (11), suggesting that 3D-CTBA improves the safety and efficacy of thoracoscopic segmentectomy and lobectomy (11). In this way, 3D-CTBA can effectively guide thoracoscopic segmentectomy and ensure the safety of surgery, especially when performing difficult segmentectomy (12).

The right upper lobe (RUL) is an area with a high incidence of pulmonary nodules. A comprehensive understanding of anatomical types of vessels and bronchi in the RUL, as well as rare variations, are essential to improve the surgical procedure and safety (13). Because the RUL is small, even if the nodule location is deep, wedge resection can usually be performed. But at the same time, a large wedge resection often severely affects the function of two lung segments, making wedge resection of the RUL controversial, leaving considerable room for segmentectomy. In the present study, the clinical data of patients undergoing thoracoscopic RUL segmentectomy were retrospectively analyzed to compare the safety and effectiveness of patients who underwent preoperative 3D-CTBA with those who underwent regular CT imaging.

## Material and methods

### Ethics statement

The present study was approved by the Institutional Review Board of the Fourth Hospital of Hebei Medical University (2021KS029), and the requirement for patient informed consent for inclusion was waived because of the retrospective nature of the study.

### Study designs and patients

This study retrospectively included patients with pulmonary nodules undergoing thoracoscopic RUL segmentectomy at the Thoracic Surgery Department of the Fourth Hospital of Hebei Medical University from December 9, 2013, to June 2, 2021. Inclusion criteria were as follows: (1) chest CT revealed a solitary ground-glass nodule of the RUL, with a lesion diameter ≤2 cm, ground-glass composition ≥50%, and doubling time ≥400 days; (2) chest CT suggested a solid nodule, which was a highly suspicious benign one with a diameter of ≤2 cm, or could not be entirely excluded as a solitary metastasis with a diameter of ≤2 cm; (3) the nodules were in the periphery of the upper lobe of the right lung, and it was difficult to perform a wedge resection. The exclusion criteria were as follows: (1) cardiopulmonary dysfunction and unable to tolerate one-lung ventilation; (2) severe liver and kidney dysfunction and cardio-cerebrovascular diseases; (3) neoadjuvant chemoradiotherapy before surgery.

### Preoperative 3D reconstruction

Preoperatively, a thin-slice enhanced CT scan of the pulmonary nodule was completed; the DICOM (digital imaging and communications in medicine) format data of the CT image was copied and imported into the Mimics 19–21.0 (Materialise's interactive medical image control System 19–21.0) software for 3D-CTBA to reconstruct the bronchi, pulmonary arteries and veins, and pulmonary nodules, which were distinguished by being highlighted different colors; then, the surgeon simulated the procedure based on the 3D reconstructed image and developed the optimal regimen.

### Thoracoscopic surgery

Under general anesthesia, double-lumen endotracheal intubation and one-lung ventilation were performed, and the patient was in the left lateral position at 90°. A thoracoscope port was made at the sixth or seventh intercostal space of the midaxillary line with an incision of 1.0 cm. The surgical port was at the third or fourth intercostal space of the anterior axillary line with an incision of 3–4 cm. Most patients underwent two-port video-assisted thoracic surgery (VATS) for lung segment surgery of the upper lobe of the right lung, but in several cases, an auxiliary surgical port had to be added due to the difficulty of the operation, which was about at the eighth intercostal position on the posterior axillary line. For the 3D reconstruction group, the 3D-CTBA reconstructed image was displayed on a separate monitor placed near the endoscopic monitor display that was easy for the surgeon to observe. The intraoperative automatic control 3D-CTBA image controller was put into a sterile bag. Intraoperatively, the surgeon could rotate the 3D-CTBA display or hide the 3D reconstructed image in real time as needed. No such 3D-CTBA images were available in the study period of the control group. Under thoracoscopy, the pulmonary arteries, bronchi, and veins of the lesion were sequentially dissected, and a linear cutting stapler (Johnson / Johnson, USA) was used for successive cutting. After the bronchus and target segment artery was cut [linear cutting stapler for severed artery: stapler line 35 mm, nail height 2.5 mm; for linear cutting stapler for severed bronchus: stapler line 60 mm, nail height 3.8 or 3.6 mm (selected according to the thickness of the bronchus); linear cutting stapler lung tissue: stapler line 60 mm, nail height 3.8], the anesthesiologist was told to inflate the lungs completely, and the “inflation–deflation method” was used for 10–15 min to identify the boundary between the lung segment of the lesion and the remaining lung segments. In this method, after the target segment bronchus is cut, the lung is inflated, and then one-lung ventilation is performed (14). After simple dissociation with an electrical hook along this boundary, a linear cutting stapler was used to remove the lesion. Lymph node sampling at N1 and N2 stations was conducted. After deflation of the remaining lung segments for about 10–15 min, the target segment lung continues to be inflated, and a boundary is formed between the deflated lung tissue and the inflated target segment lung tissue, thereby determining the intersegmental interfaces. During the operation, after the target lung tissue was removed and taken out, a physiological sodium chloride solution was injected into the thoracic cavity through the operation hole, and the anesthesiologist was instructed to inflate the lung (to a pressure of ≤20 cmH_2_O) to determine whether the remaining lung was well inflated and whether there was air leakage. If there was no obvious or slight air leakage, it was not handled. In case of obvious air leakage, 3-0 Prolene suture was applied first, and then medical glue was sprayed on the surface (*α*-cyanoacrylic acid fast medical adhesive). Then, a chest tube was indwelled in the observation port, and the operating port was sutured.

The indications for single-segment resection were (1) the nodules were small and especially close to the periphery, or the nodules could be specifically determined as benign; (2) due to the uneven development of each segment, sometimes one segment is particularly hypertrophic (dominant lung segment, such as S3b in this study); and (3) the anatomy of individual subsegments is particularly convenient for single-segment resection. The procedure is basically the same as that of lung segment resection. The target segment blood vessels and subsegment bronchus were dissected. The plane between subsegments was determined using the expansion and collapse method. The plane was separated using an electric hook and a linear cutting occluder.

### Outcomes and data collection

The main outcome measures included operation time, intraoperative blood loss, drainage duration and drainage volume, hospital stay, and postoperative complications [pulmonary infection, atrial fibrillation, and deep vein thrombosis]. The demographic characteristics, pathological findings (maximum tumor diameter, histopathological type, and pathological TNM staging), and intraoperative and postoperative data were collected. Intraoperative blood loss = blood volume in the drainage bottle through the suction device + blood absorption volume of gauze (estimated at 20 ml/piece of gauze).

### Statistical methods

Statistical analyses were performed on SPSS 23.0 software (IBM Corp., USA). Normally distributed measurement data were presented as mean ± standard deviation, and the independent-samples *t*-test was used for comparison between groups; data of skewed distribution were presented as median (interquartile range, IQR) and compared by the Mann–Whitney *U* test. Count data were presented as absolute numbers or percentages and compared by the chi-square test or Fisher's exact test. The risk factors for intraoperative bleeding and operation time were analyzed by linear regression. *P* *< *0.05 was considered statistically significant.

## Results

### Baseline characteristics of patients

**Figure 1:**
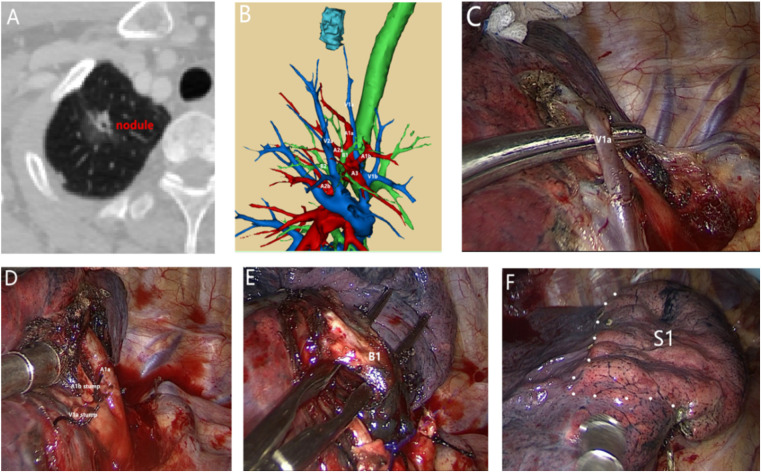
A 54-year-old male patient with nodules in the upper lobe of the right lung and a history of diabetes underwent right upper lobe apical segment resection. Postoperative pathology revealed invasive adenocarcinoma. (**A**) Preoperative computed tomography, right upper lobe apical nodules, 1.7 cm in diameter. (**B**) Preoperative 3D bronchovascular imaging of the right upper lobe apex segment. (**C**) Separation of V1a. (**D**) Separation and detachment of A1a and A1b. (**E**) Separation of B1. (**F**) S1 intersegment plane.

A total of 139 patients were enrolled in the present study, including 46 males and 93 females, with a mean age of 49.43 ± 7.227 (16–82) years and a median nodule size of 1 (0.9–1.3) cm. There were 128 patients with NSCLC (including 67 with adenocarcinoma *in situ*, 48 with minimally invasive adenocarcinoma, 10 with invasive adenocarcinoma, 2 with mucinous adenocarcinoma, and 1 with a carcinoid tumor). There were three patients with atypical adenomatous hyperplasia and four with metastases (two with breast cancer metastases, one with kidney cancer metastases, and one with rectal cancer metastases, all detected using positron emission tomography). In this study, three patients with inflammatory pseudotumor and one patient with bronchiectasis underwent S1 resection. The preoperative imaging data of three patients with benign lesions suggested that they were benign tumors. The rapid frozen examination after nodule resection indicated an inflammatory pseudotumor. The patient with bronchiectasis had acute bronchiectasis with pulmonary bullae and underwent S1 resection. In the whole group, three patients (2.2%) underwent single segmental resection, including S1A, S2A, and S3b. Of all patients, 85 were treated from 2018 and, therefore, underwent 3D-CTBA (3D-CTBA group). The 54 patients treated before 2018 did not undergo 3D-CTBA (non-3D-CTBA group). When the baseline characteristics were compared for the two groups, there were no statistically significant differences between the two groups in age, gender, smoking, comorbidities, tumor size, or tumor type (Table 1). Figure 1 presents a typical case with nodules in the upper lobe of the right lung and a history of diabetes who underwent right upper lobe apical segment resection.

**Table 1:** Comparison of baseline data between the two groups of patients who underwent segmentation of the right upper lobe.

Characteristic	Non-3D-CTBA group (*n* = 54)	3D-CTBA group (*n* = 85)	*P*
Age (y), mean ± SD	49.1 ± 5.3	49.6 ± 8.2	0.668
Sex (Male/Female)	17/37	29/56	0.748
Smoking, *n* (%)	22 (40.7)	29 (34.1)	0.430
Comorbidities, *n* (%)			
Chronic COPD	14 (25.9)	18 (21.2)	0.517
Hypertension	8 (9.3)	19 (22.4)	0.274
Diabetes	7 (13.0)	8 (9.4)	0.511
Coronary artery disease	3 (5.6)	3 (3.5)	0.677
Cerebrovascular disease	1 (1.9)	5 (5.9)	0.405
Chronic renal failure	2 (3.7)	2 (2.4)	0.642
Arrhythmia	7 (13.0)	19 (22.4)	0.166
History of malignant tumors	7 (13.0)	11 (12.9)	0.997
Tumor size (cm), median (IQR)	1 (0.9–1.3)	1.1 (1–1.3)	0.598
Non-small-cell lung cancer, *n* (%)	49 (90.7)	78 (91.8)	>0.999
Adenocarcinoma *in situ*	24 (44.4)	42 (49.4)	
Minimally invasive adenocarcinoma	18 (33.3)	30 (35.3)	
Invasive adenocarcinoma	5 (9.3)	5 (5.9)	
Mucinous adenocarcinoma	2 (3.7)	0	
Carcinoid	0	1 (1.2)	
Atypical adenomatous hyperplasia	1 (1.9)	3 (3.5)	
Metastasis, *n* (%)	2 (3.7)	2 (2.4)	
Breast cancer metastasis	1 (1.9)	1 (1.2)	
Kidney cancer metastasis	0	1 (1.2)	
Rectal cancer metastasis	1 (1.9)	0	
Benign tumor, *n* (%)	2 (3.7)	2 (2.4)	
Inflammatory pseudotumor	2 (3.7)	1 (1.2)	
Bronchiectasis	0	1 (1.2)	
FEV1	2.54 ± 0.57	2.42 ± 0.41	0.170
FEV1/pre	87.8 (79.9–94.3)	92.1 (80.6–102.0)	0.102
Causes for segmentectomy, *n* (%)			0.521
Planned	54 (100.0)	83 (97.6)	
Unplanned	0	2 (2.3)	
Surgical procedure, *n* (%)			
S1	18 (33.3)	14 (16.5)	
S1 + S2	2 (3.7)	3 (3.5)	
S1 + S3	0	1 (1.2)	
S1 + S2a	1 (1.9)	1 (1.2)	
S1a	0	1 (1.2)	
S1a + S2a	3 (5.6)	6 (7.1)	
S1b + S3b	0	1 (1.2)	
S2	17 (31.5)	22 (25.9)	
S2 + S1a	0	4 (4.7)	
S2a	1 (1.9)	0	
S2b + S3a	2 (3.7)	17 (20.0)	
S3	9 (16.7)	14 (16.5)	
S3 + S1b	1 (1.9)	0	
S3b	0	1 (1.2)	

COPD, chronic obstructive pulmonary disease; 3D-CTBA, three-dimensional computed tomography bronchography and angiography; IQR, interquartile range.

### Comparison of perioperative results between the two groups

The operation time of the 3D-CTBA group was significantly shorter than that of the non-3D-CTBA group (133.4 ± 20.9 vs. 143.0 ± 10.8, *P* = 0.001), and intraoperative blood loss was significantly lower than that of the non-3D-CTBA group (67.4 ± 17.6 vs. 73.1 ± 11.0, *P* = 0.021) (Table 2). In patients undergoing S1 segmentectomy, the operative time (119.4 ± 12.4 vs. 137.4 ± 7.0 min, *P* < 0.001) and intraoperative blood loss (56.6 ± 12.4 vs. 67.9 ± 7.2 ml, *P* = 0.003) of the 3D group were also significantly lower than that of the non-3D group. However, there were no significant differences in operative time and intraoperative blood loss in patients undergoing S2 or S3 segmentectomy.

**Table 2:** Comparison of perioperative results between the two groups of patients who underwent segmentation of the right upper lobe.

Characteristic value	Non-3D-CTBA group (*n* = 54)	3D-CTBA group (*n* = 85)	*P*
Operation time (min), mean ± SD	143.0 ± 10.8	133.4 ± 20.9	0.001
Intraoperative blood loss (ml), mean ± SD	73.1 ± 11.0	67.4 ± 17.6	0.021
Postoperative complications, *n* (%)			
Lung air leakage	7 (13.7)	4 (4.7)	0.108
Pneumonia	6 (11.1)	6 (7.1)	0.537
Hemoptysis	1 (1.9)	0	0.388
Atelectasis	2 (3.7)	2 (2.4)	0.642
Extubation time (d), median (IQR)	5 (4.8–6)	5 (5–6)	0.732
Discharge time (d), median (IQR)	7 (6–8)	7 (6–8)	0.735

3D-CTBA, three-dimensional computed tomography bronchography and angiography; IQR, interquartile range.

### Analysis of risk factors for intraoperative blood loss and operation time

Multivariate analysis revealed that age and gender were not risk factors for intraoperative blood loss, but 3D-CTBA (Beta = −7.594, 95% CI: −12.877 to −2.311, *P* = 0.005) and surgical procedure (Beta = 9.352, 95% CI: 3.551 to −15.153, *P* = 0.002) were independently associated with intraoperative blood loss (Table 3). 3D-CTBA (Beta = −13.027, 95% CI: −18.632 to 17.422, *P < *0.001) and surgical procedure (Beta = 7.072, 95% CI: 0.864–13.280, *P* = 0.026) were independent factors affecting the operation time (Table 4).

**Table 3:** Univariate/multivariate analyses of risk factors for intraoperative blood loss in patients who underwent segmentation of the right upper lobe.

Characteristic	Univariate analysis			Multivariate analysis		
	Beta	95% CI	*P*	Beta	95% CI	*P*
Age	−0.311	−0.672 to −0.05	0.090			
Sex (male vs. female)	−1.501	−7.073 to −4.071	0.595			
COPD	−0.554	−6.788 to −5.680	0.861	0.006	−5.989 to −6.001	0.998
3D-CTBA (yes vs. no)	−5.679	−10.977 to −0.381	0.036	−7.594	−12.877 to −2.311	0.005
Surgical procedure (segmentectomy vs. subsegmentectomy)	7.492	1.741 to 13.242	0.011	9.352	3.551 to 15.153	0.002

COPD, chronic obstructive pulmonary disease; 3D-CTBA, three-dimensional computed tomography bronchography and angiography; CI, confidence interval.

**Table 4:** Univariate/multivariate analyses of factors affecting the operation time for patients who underwent segmentation of the right upper lobe.

Characteristic	Univariate analysis			Multivariate analysis		
	Beta	95% CI	*P*	Beta	95% CI	*P*
Age	−0.414	−0.835 to −0.007	0.054			
Sex (male vs. female)	−4.344	−10.831 to −2.143	0.188			
COPD	0.203	−7.094 to −7.501	0.956	−0.491	−6.918 to −5.937	0.880
3D-CTBA (yes vs. no)	−9.598	−15.683 to −3.508	0.002	−13.027	−18.632 to −17.422	<0.001
Surgical procedure (segmentectomy vs. Subsegmentectomy)	4.254	−2.602 to 11.109	0.222	7.072	0.864 to 13.280	0.026

COPD, chronic obstructive pulmonary disease; 3D-CTBA, three-dimensional computed tomography bronchography and angiography; CI, confidence interval.

## Discussion

The results showed that 3D-CTBA cases had decreased intraoperative blood loss and shorter operation times. 3D-CTBA and surgical procedures were independently associated with intraoperative blood loss, and these two factors were also independent factors affecting the operation time. Therefore, this study supports the use of 3D-CTBA preoperatively for anatomical segmentectomy of the RUL with clear benefits to the patients in terms of less blood loss and shorter operation times.

For the 3D reconstruction group, the 3D-CTBA reconstructed 3D image was displayed on another screen placed beside the operation screen, and it was easy for the operator to observe the 3D-CTBA next to the endoscopic display. The intraoperative automatic 3D-CTBA image controller was placed into a sterile bag. The intraoperative operator could rotate, display, or hide the 3D-CTBA reconstructed image in real time according to the operation requirements and carry out the operation in real time. This setting could be conducive to improving intraoperative navigation.

Previous studies have suggested that 3D-CTBA would be helpful for pulmonary segmentectomy (15–17), but few studies compared 3D-CTBA with the results of patients evaluated with 2D-CT. Hagiwara et al. showed that patients undergoing thoracoscopic segmentectomy or lobectomy guided by 3D-CTBA have a lower incidence of postoperative complications and a significantly shorter operation time (11). Another study by Oizumi et al. (12) also suggested that 3D-CTBA can effectively guide thoracoscopic segmentectomy and ensure the safety of surgery, especially when performing difficult segmentectomy. These findings indicate that the preoperative use of 3D-CTBA to assess pulmonary vascular branching patterns seems to improve the safety and efficacy of thoracoscopic segmentectomy and lobectomy. This may prove to be a major benefit to patients who are increasingly undergoing resection of complex segments. The results of this study showed that the blood loss during the procedure was smaller with the use of 3D-CTBA. Liu et al. (18) collected the clinical data of 124 patients who underwent thoracoscopic pulmonary segmentectomy and were allocated to three groups: the general group, the 3D-CT group, and the 3D printing group. Compared with the general group, intraoperative blood loss in the 3D-CT group and 3D printing group decreased significantly. Operation time in the 3D-CT group and 3D printing group was significantly shorter than in the general group. Between the 3D-CT group and 3D printing group, intraoperative blood loss and operation time had no significant differences. Postoperative chest tube duration and postoperative hospital stay had no significant differences between each group, which were consistent with the findings of the present study. However, another study suggested that 3D-CTBA reduced intraoperative and postoperative complications compared with 2D VATS (19). Postoperative short-term complications are an important indicator for evaluating segmentectomy. In recent years, the complication rate of VATS-based segmentectomy has been reported to be 11.5%–34.9% (20). The findings in this study showed that no perioperative deaths occurred, and postoperative complications occurred in 25 (18.0%) cases, mainly involving lung air leakage, pneumonia, hemoptysis, or atelectasis, and all were resolved during hospitalization. Regarding the reason for less bleeding in the 3D group, it could be because of the preoperative planning, the surgeons had a better grasp of the patient's anatomy during the operation, and the anatomical operation level was more clearly during the operation. The targeted blood vessels could be separated purposefully, and the damage to other tissues was less.

There is a suggestion that as imaging and techniques are developed, more complex segmentectomy methods are being used, so here we also present a comparison of our clinical data and techniques with other similar studies. In a 2-year learning experience study by Duan *et al.* (21) involving 156 patients undergoing single-port thoracoscopic segmentectomy, a total of 156 segmentectomies were performed, including S1 (*n* = 14), S2 (*n* = 16), and S3 (*n* = 6). In the present study, 93 (66.9%) completed single segmentectomy, including S1 (*n* = 32), S2 (*n* = 38), and S3 (*n* = 23). Undoubtedly, single-segment resection is relatively easy and predominant in anatomical sublobar resection. Among the three segments, it seems that the posterior segment resection is relatively easy, so it accounts for the highest proportion in the present study. Due to the anatomical position and adjacent structures, the anterior segment resection seems to be more difficult, so it has a lower proportion. In a study by Wu *et al.* (22), among 146 undergoing thoracoscopic cone-shaped segmentectomy, there were 43 undergoing subsegmentectomy and 38 combined segmentectomy. Generally, single subsegmentectomy of the RUL is rarely performed because the RUL has six subsegments, and the volume of one subsegment is even smaller than a normal wedge resection, so the boundary is difficult to be determined. Regarding single subsegmentectomy, three (2.2%) completed single subsegmentectomy, namely S1a, S2a, and S3b. Due to the anatomical position, many subsegments seem to be difficult to remove, such as S2b and S3a. Generally, this procedure is suitable for smaller nodules, particularly close to the periphery, or for confirmed benign nodules.

The inflation–deflation method was adopted to determine the resection plane. This method is simple and easy, requires no special drugs or equipment, and allows direct visualization of the area to be resected. As long as the target segment artery is disconnected correctly, the intersegment plane can be clearly found by this method, as previously described (23–25). Indocyanine green requires fluorescence thoracoscopy equipment; according to the preoperative 3D reconstruction results, the bronchus, pulmonary artery, and pulmonary vein of the target segment can be accurately identified and separated (26, 27). Still, if complicated pulmonary segment resection is encountered, the target segment artery cannot be clearly identified, or if there are communicating branches and variant branches of other segment arteries in the target segment, it will lead to plane positioning deviation between segments. Moreover, although it is also a promising method, the quantitative analysis of indocyanine green imaging results needs to be further explored and confirmed, and allergic reactions were reported (26–29). In addition, the fluorescence of the indocyanine green counterstaining method can only be developed on the visceral pleura between segments, and the parenchymal part of the interface between segments cannot be developed (26–28). Indocyanine green injected into the target segment stump bronchus can effectively identify the parenchymal intersegmental plane through indocyanine green navigation (30), but the operation is not so simple and requires high experience from the operator, with a steep learning curve.

This study has some limitations. This was a single-center retrospective study with a small sample size. There was no direct comparison between treatment groups undergoing treatment at the same time. As techniques and experience in segmentectomy develop, outcomes might improve, so the difference in treatment time between the two groups may have introduced some bias into the study. A larger sample size from multiple centers would add more evidence to support these results.

## Conclusion

Preoperative use of 3D-CTBA to evaluate and simulate pulmonary blood vessels and bronchial branch patterns is conducive to the safe and effective performance of thoracoscopic segmentectomy. Because the RUL has a regular arrangement and its anatomy is not too complicated, it may be more suitable for anatomical sublobar resection than other parts of the lung, and this approach is worthy of vigorous development.

## Data Availability

The original contributions presented in the study are included in the article/Supplementary Material, further inquiries can be directed to the corresponding author.
